# Special Meeting of the “Chicago Society of Physicians and Surgeons,” Monday Evening, August 18, 1873

**Published:** 1873-10

**Authors:** Plymmon S. Hayes

**Affiliations:** Chicago


Article III.—*Special Meeting of the "Chicago Society of Physicians and Surgeons," Monday evening, August* 18, 1873. Reported by Plymmon S. Hayes, M.D., Chicago.

The meeting was called for the purpose of continuing the discussion of the subject of cholera. Dr. Owens was called to the chair.

Dr. Simons made the following verbal report: There had been ten deaths from cholera since the last meeting. The temporary hospital, situated near Burnside street, between 33rd and 35th streets, had been opened Tuesday, August 12th. A number of patients had been admitted, and these, when not in a stage of collapse at or about the time of their removal, appeared to enter upon convalescence more speedily than when treated at their residences. Carbolic acid, sulphate of lime, and chloride of iron, had been largely used as disinfectants. Five cases were in the hospital at date, and all but one were progressing favorably. Two of the nurses had exhibited premonitory symptoms of the disease, but soon recovered.

Little attention had been paid by the inhabitants of the infected district to the circulars distributed by the Board of Health. Even when summoning a physician to patients in collapse, they generally declared that the existing disorder was nothing graver than a simple diarrhœa.

One-third of the inhabitants had left the infected locality, and in spite of all remonstrance, few of the Germans could be induced to destroy their feather beds upon which cholera patients had died. Upon removal they generally carried with them these beds, and such elements of contagion as were capable of transportation therein.

Dr. Wood had examined under the microscope some water obtained from a filter well at No. 947 Burnside street, south of the city limits, and presented a specimen of it to the Society. The well was ten feet below the surface, and contained, therefore, surface water merely. The water had been filtered through two layers of sand and one of charcoal. Upon applying the permanganate of potassa test, he estimated that it contained 91,279 grains of oxidizable organic matter to the gallon, consisting mostly of a disorganized vegetable detritus, evident under the microscope.

Dr. Simons remarked that the majority of the recent cases had not made use of well water, but of hydrant water. Public hydrants had been that day established as far south as 37th street, the city limits, and the hydrant water had thus been placed within easy access of the entire neighborhood.

Dr. Boyd stated that the first cases of cholera in the section referred to, occurred in a family which had resided there for two years—the father and son were laborers, and their fare was extremely meagre. Every member of the family died; and he considered that every subsequent case was more or less directly traceable to this source of infection.

Dr. Wilder reported a case on 20th street, which Dr. Chas. Gilmore Smith considered to be a case of death from dysenteric hemorrhage.

Dr. Hyde read a letter from Dr. Davis Halderman, of Columbus, Ohio, giving details of fifty cases of cholera with twenty-seven deaths. The disease first appeared just outside the penitentiary, and the cases were confined to those living on low grounds. No symptoms of the disease had occurred in the infirmary, where, for several reasons, it might have been expected.

The speaker had secured a "rice-water stool" from Dr. Boyd, and had forwarded it under seal to Dr. I. N. Danforth for microscopical examination. The latter had just telegraphed that he had been unavoidably detained from attendance.

On motion, it was resolved that Dr. Danforth be requested to make known the results of his researches at the next meeting.

Dr. Simons stated that the following formula, obtained from Dr. T. Bevan, a member of the Society, had been employed with success:

R. Acid Sulphurosi, - - - - - - oz. j.
Magnes. Sulphitis, - - - - - dr. j.
Capsici Tincturæ, - - - - - oz. ss.
Aquæ Destillat., - - - - - oz. j.
M. et S. Cochl. parv. no. j. in cyath. aq. bis die.

This preparation was intended as a prophylactic. When used as a remedy, two grains of morphia were added to the above, and a teaspoonful exhibited in water every half hour.

The following committees were then appointed, with instructions to report fully as soon as practicable:

"*Committee upon the history, nature and contagious character of the disease existing at present near the southern borders of the city.*"

DR. THOMAS BEVAN, *Chairman.*

DR. JNO. BARTLETT. DR. I. N. DANFORTH.

DR. C. J. SIMONS. DR. LESTER CURTIS.

"*Committee on the most appropriate prophylaxis and treatment of the disease.*"

DR. J. H. ETHERIDGE, *Chairman.*

DR. H. P. MERRIMAN. DR. U. M. BOYD.

DR. CHAS. GILMORE SMITH. DR. F. H. DAVIS.

On motion, it was requested that hereafter the Secretary of the Society supervise the reports prepared for publication of its transactions in the secular journals.

The Society then adjourned.

